# R990G Polymorphism of Calcium Sensing Receptor Gene Is Associated with High Parathyroid Hormone Levels in Subjects with Vitamin D Deficiency: A Cross-Sectional Study

**DOI:** 10.1155/2015/407159

**Published:** 2015-01-28

**Authors:** Hafsa Majid, Aysha Habib Khan, Tariq Moatter

**Affiliations:** ^1^Section of Chemical Pathology, Department of Pathology & Microbiology, Aga Khan University, Stadium Road, Karachi 74800, Pakistan; ^2^Section of Molecular Pathology, Department of Pathology & Microbiology, Aga Khan University, Stadium Road, P.O. Box 3500, Karachi 74800, Pakistan

## Abstract

Single nucleotide polymorphisms (SNPs), R990G and A986S of the calcium sensing receptor (CaSR) gene, are shown to influence response of parathyroid hormone (PTH) in subjects with optimal vitamin D levels. This cross-sectional study was conducted in subjects with vitamin D deficiency (VDD) to observe associations between CaSR polymorphisms, plasma iPTH, and serum calcium levels. Adult females (*n* = 140) with known VDD, intact parathyroid hormone (iPTH), and calcium levels were recruited for genotype analysis. The frequencies of the 986 alleles GG, GT, and TT were 68%, 25%, and 7%, respectively, whereas the frequencies of the 990 alleles AA, AG, and GG were 80%, 8.9%, and 11.1%, respectively. The subjects with GG genotype of R990G polymorphism had higher iPTH levels (148.65 versus 91.47 and 86.1 pg/mL for GG versus AA, AG, resp., *P* = 0.008) and lower calcium levels (8.4 versus 9.04 and 9.07 mg/dL for GG versus AA, AG, resp., *P* = 0.002). No such association of A986S polymorphism with plasma iPTH or serum calcium levels was observed in the present study. Patients with VDD bearing the GG genotype of R990G SNPs are prone to have higher iPTH levels and lower calcium.

## 1. Introduction

Vitamin D deficiency has been reported from Pakistan in healthy volunteers, patients, and community females [1–3]. In those studies, a variable proportion (25.9%–31%) of the subjects demonstrated secondary hyperparathyroidism (SHPT), as a compensatory response to VDD. On the contrary, a significant number of subjects with VDD did not show SHPT (blunted PTH response), a phenomenon described in literature as functional hypoparathyroidism [4]. Several reasons have been reported for the inconsistent PTH response to VDD. Factors causing functional hypoparathyroidism include abnormalities of the CaSR or vitamin D receptor, magnesium deficiency, and renal dysfunction [4, 5]. The focus of our study is the CaSR.

The main physiological function of parathyroid gland is maintaining blood calcium levels within a narrow range, by modulating minute-to-minute release of PTH into the circulation.

The CaSR has been shown to play a key role in calcium homeostasis by regulating PTH secretion. It is a G-protein coupled receptor, which is expressed in many cell types associated with maintaining calcium homeostasis, including parathyroid cells, kidney, and osteoblasts [6]. Patients with functional hypoparathyroidism show a minimum secretion rate of PTH in the presence of low or low normal calcium as compared to subjects with SHPT. This indicates abnormal calcium sensing which can occur due to changes in genetic makeup of CaSR. Recent studies have shown association of SNPs in CaSR gene with serum levels of PTH and calcium [7–9]; small increments in extracellular calcium above normal would lead to activation of CaSR which prevents immediate secretion of PTH, gene transcription, and proliferation of parathyroid cells [10, 11].

Several SNPs regulating CaSR gene are reported in the Caucasians; of those A986S and R990G are most frequently studied. These variant alleles have been studied in different groups including normal individuals, subjects with primary hyperparathyroidism, and kidney stone formers [12, 13]. To the best of our knowledge, there is no published report exploring the association of CaSR linked SNPs with SHPT in subjects with VDD.

We hypothesized that variable PTH secretion in VDD subjects is influenced by the SNPs located in the CaSR gene. Therefore, this study was conducted to explore an association between SNPs A986S and R990G with PTH and calcium levels in Pakistani females confirmed with VDD.

## 2. Materials and Methods

### 2.1. Study Design and Population

A cross-sectional study was conducted between January 2012 and September 2012 in the Sections of Chemical and Molecular Pathology, Department of Pathology and Microbiology, Aga Khan University, Karachi, Pakistan. A total of 140 premenopausal female subjects, ≥18 years of age (age range 18–48 years) with VDD (25 OHD level < 20 ng/mL), were analyzed for SNPs in the CaSR gene. Subjects with any medical disorder of bone minerals, such as primary hyperparathyroidism or hypoparathyroidism, were excluded from the study. Females taking any medications known to affect calcium metabolism, such as calcium tablets or bisphosphonate, and vitamin D supplements, individuals on hormonal therapy, and pregnant females were also excluded. The study was approved by ethical review committee of the Aga Khan University Hospital (ERC approval number: 2155-Path-ERC-12).

### 2.2. DNA Extraction and Genotyping Procedure

Genomic DNA was extracted from WBC obtained from blood specimens of the participants using a DNA isolation kit, according to the manufacturer's instructions (Promega, Madison, WI, USA). Purified DNA was stored at −20°C for further processing.

The SNPs A986S and R990G located in the exon 7 of CaSR gene were analyzed by RFLP-PCR using published protocols [14]. A986S polymorphism represents substitution of alanine with serine (replacement of nucleotide G with T) at position 986, whereas R990G polymorphism is substitution of arginine with glycine (replacement of nucleotide A with G) at position 990. Restriction enzymes Hsp-921 (Promega, Madison, WI, USA) and Btg-I (New England Biolabs, Hitchin, UK) were used for the analysis of A986S and R990G polymorphisms, respectively. Restriction assay was performed in a total volume of 25 *μ*L containing 15 *μ*L PCR product, 3 *μ*L buffer, 0.2 *μ*L BSA, 11.3 *μ*L nuclease free water, and 5 units of the specific enzyme. Reaction mix was incubated overnight in water bath at 37°C. Restriction products were then analyzed by 3% agarose gel electrophoresis. A986S genotypes included GG, GT, and TT, whereas R990G genotypes were AA, AG, or GG. All assays were examined with appropriate controls.

### 2.3. Biochemical Analysis

Serum 25-OHD levels were measured by electrochemiluminescence immunoassay on Elecsys auto analyzer (Roche Diagnostics, Basel, Switzerland). For quality assessment, low, medium, and high Elecsys Preci Controls were used. The within-run CVs were 5.7%, 5.7%, and 5.4% at concentrations of 25.2, 39.9, and 65.6 ng/mL. A cutoff of <20 ng/mL for 25OHD was considered to be deficient. Plasma intact parathyroid hormone (iPTH) levels were measured by Immulite 2000 (Siemens Healthcare Diagnostics Inc., NY, USA). Two levels manufacturer provided controls were run for quality assessments. The intra-assay precision was 5.5%; an interassay precision was 7.9%. The reference range of iPTH assay was 16–87 pg/mL. Plasma iPTH levels above 87 pg/mL were considered as hyperparathyroidism, while SHPT was defined as subjects with low or normal calcium and deficient 25OHD levels and PTH >87 pg/mL.

Serum calcium levels were measured on Advia 1800 Chemistry Analyzer (Siemens Healthcare Diagnostics Inc., NY, USA). Two levels manufacturer provided controls were run for quality assessments. For the period of study, external quality was assured by simultaneously analyzing samples for 25OHD, iPTH, and calcium from College of American Pathologist (CAP, USA) three times a year.

### 2.4. Data Analysis

Data was analyzed by statistical package for social sciences (SPSS) version 19 for Windows (IMB, Chicago, IL, USA). For quantitative variables (age, BMI, creatinine, calcium, iPTH, and vitamin D levels), mean and standard deviation were calculated. Distribution of SNPs was compared with iPTH, calcium, and 25OHD levels using one way analysis of variance, taking R990G and A986S as dichotomizing factors and iPTH, calcium, and 25OHD as dependent variables. Online software SNPstats [15] was used to perform Fisher's exact test for computing Hardy-Weinberg equilibrium and association analyses for iPTH, calcium, and 25OHD levels with the different haplotypes.

## 3. Results

The baseline biochemical data of the 140 women enrolled in the study is shown in Table 1. All the participants were vitamin D deficient. The mean creatinine and calcium levels were within normal limits. According to South Asian Classification, most of the women were overweight. The mean iPTH level of the participants was slightly higher compared to the upper limit of the reference range used; of them 45% of the women had raised iPTH (mean iPTH 145.3 ± 9.5 pg/mL) and 55% had iPTH levels within reference range (mean iPTH 59.9 ± 17.2 pg/mL).

### 3.1. Frequency of A986S and R990G Genotypes

The allelic frequency of A986S SNPs for nucleotides G and T was 76% and 24%, respectively. Genotype GG of SNP A986S was most frequent (68%) in the study group, followed by GT (25%) and TT (7%). The A986S genotype incidence was in accordance with the Hardy-Weinberg equilibrium (Fisher's exact test, *P* = 0.46). Allelic frequency for R990G SNPs for nucleotides A and G was 72% and 28%, respectively. The frequency of genotype AA of R990G was observed in 80% participants, whereas the genotypes AG and GG were found in 9% and 11% of women, respectively. However, these genotypes were not in Hardy-Weinberg equilibrium (*P* value < 0.0001).

### 3.2. Effects of SNPs on iPTH and Calcium Levels

Tables 2 and 3 show analysis of calcium, iPTH, and 25OHD levels grouped according to A986S and R990G genotypes, respectively. Comparison of A986S genotypes showed no statistically significant difference in three genotypes for iPTH, calcium, and vitamin D.

Comparison of participants among the three genotypes of R990G SNP revealed that serum iPTH levels are significantly higher in GG compared to AG and AA genotypes (148.65 versus 90.39 and 86.1 pg/mL, *P* = 0.008). In addition, decreased calcium levels (8.47 versus 9.07 mg/dL, *P* = 0.002) were seen in subjects with GG genotype, while statistically nonsignificant difference in 25OHD levels within the three genotypes was noted.

The commonest R990G:A986S haplotype found in our population was A:G. The quantitative variables were compared in individuals with different haplotypes, taking A:G as the reference group (Table 4). Intact PTH levels were significantly higher in individuals carrying G:G haplotypes when compared with A:G haplotype subjects (mean PTH 132.34 ± 118.5 pg/mL versus 86 ± 47.5 pg/mL, *P* = 0.0048). Serum calcium concentration was significantly lower in individuals carrying haplotype G:T (mean calcium 9.05 ± 0.48 versus 8.27 ± 1.96 mg/dL, *P* = 0.0079) compared to participants with haplotype A:G.

Decreasing levels of calcium were found in haplotypes observed in the following order: A:T > A:G > G:G > G:T, while gradual iPTH increase was seen in the following haplotypes: G:T > G:G > A:T > A:G.

## 4. Discussion

Analysis of genetic polymorphisms associations and disease states or alterations in physiological and other variables can provide clues into disease pathogenesis, which can be explored further by experimental approaches. There have been a number of studies examining associations of CaSR polymorphisms with variables such as serum calcium concentrations, PTH levels, severity of primary hyperparathyroidism, calcium excretion, renal stones, fractures, bone mineral density, and risk of colon cancer [8, 12, 13, 16]. All of these are conducted in vitamin D sufficient individuals; so far there is no study reported in literature that has reported association of CaSR polymorphisms with PTH and calcium levels in subjects with VDD.

In parathyroid tissue, vitamin D and calcium have been recognized as key modifiers of PTH gene transcription, hormone synthesis, and parathyroid cell proliferation. Recent literature shows that all the effects of extra cellular calcium are mediated through CaSR [6]. The role of CaSR in parathyroid tissue extends beyond its traditional role as a modifier of calcium regulated PTH secretion and involves other components of parathyroid gland function which are frequently abnormal in clinical disorders characterized by excess parathyroid gland activity such as hyperparathyroidism. The SHPT is a normal compensatory response in VDD, which maintains serum calcium levels but at the expense of decreasing mineral content of bone, causing bone thinning and early osteoporosis especially in females [1, 17, 18]. On the other hand, subjects with functional hypoparathyroidism are shown to have higher bone mineral density compared with those having SHPT [4, 19].

The frequencies of A986S and R990G SNPs in our population were close to their distribution in Caucasians and Asian population (16.5% and 4.5% versus 7% for A986S and 8.4% and 42.9% versus 11.1% for R990G SNP, resp.) [20]. Major aim of the present study was to investigate whether a given genotype has a role in determining the interrelationship between levels of iPTH, calcium, and vitamin D. The GG genotype of R990G SNP was found to be associated with higher mean iPTH levels. Only 16 subjects were identified with GG genotype of R990G polymorphism and 12 out of 16 subjects had higher than normal mean iPTH levels. Since the degree of the disequilibrium was small, therefore, the small number of homozygotes could have confounded interpretation of the genotype-analyte association. Subjects with GG genotype of R990G polymorphisms had lower mean calcium levels despite SHPT. This finding could have been due to the increased excretion of calcium in subjects with R990G polymorphism, as reported in multiple studies. Subjects with vitamin D deficiency carrying the GG genotype may develop lower calcium serum levels than patients with other genotypes because patients have two hypocalcemic stimuli, the low vitamin D level and the 990G allele. PTH secretion could be secondarily stimulated in these patients.

The SHPT due to VDD is treated with calcium and vitamin D supplements. However, recently in patients on hemodialysis due to CKD [21], CaSR based therapeutics (calcimimetics) provides better therapy options by modulating PTH secretion rather than just supportive treatment [22, 23]. Calcimimetics are orally active agonists of the CaSR, used for treating PTH hypersecretion in end stage kidney disease and parathyroid carcinoma by enhancing receptor sensitivity to extracellular calcium and inhibiting PTH secretion. Several studies have shown that polymorphisms in carboxyl end terminal of CaSR gene can lead to increased sensitivity of those individuals to calcimimetics [24, 25].

In the present study, we found that the G:G haplotype is significantly associated with high iPTH levels, suggesting that it is a susceptibility marker for the development of SHPT. While the G:T haplotype was associated with lower calcium levels, whether it is exclusively mediated by a decreased inhibitory effect of the G:T haplotype of CASR on suppression of PTH secretion with subsequent decreased mobilization of calcium cannot be assessed from our data, especially given the bias due to inclusion of subjects with VDD only.

Strengths of this study are that only subjects with VDD (which is very common in Pakistan) were included and association of these polymorphisms with SHPT was determined. Accuracy of reaction was validated by using controls. To identify controls, 10 samples after similar PCR reaction and digestion controls as samples of study subject were analysed by agarose gel electrophoresis and for confirmation were rerun on 1.5% Precast Polyacrylamide Gel (Bio-Rad Laboratories Inc., USA). The samples identified as having A986S (genotype GT or TT) and R990G polymorphisms (genotype AG or GG) were then stored and run as controls with each batch of PCR reaction and electrophoresis.

Genetic and environmental covariates in specific populations can affect associational observations. The clinical significance of these polymorphisms in prediction of disorders affecting bone and mineral metabolism and homoeostasis of bone minerals needs to be explored.

In conclusion, patients with VDD bearing the GG genotype of R990G SNP have higher plasma iPTH levels and lower serum calcium in comparison with the AG and AA genotype subjects. Secondly, the presence of SHPT in our population is found to be associated with CaSR gene polymorphism.

## Figures and Tables

**Table 1:** Anthropometric and biochemical data of study subjects.

Variable (reference range)	Mean ± SD
*n* = 140	
Age (years)	31 ± 8.2

BMI (kg/m^2^)	24.2 ± 5.4

Serum 25OHD	8.4 ± 7.7
(Vitamin D deficiency <20 ng/mL)	

Plasma PTH	96.2 ± 66
(normal values 16–87 pg/mL)	

Serum calcium	9 ± 0.6
(normal values 8.6–10.2 mg/dL)	

Serum creatinine	0.8 ± 0.2
(normal values 0.6–1.2 mg/dL)	

^*^South Asian classification of BMI.

**Table 2:** Frequency and distribution of plasma iPTH, serum calcium, and serum 25OHD levels in CaSR SNP, A986S genotypes.

Analytes	A986S polymorphism			*P* value
	GG (*n* = 95)	GT (*n* = 35)	TT (*n* = 10)	
Plasma iPTH (pg/mL)	92.4 ± 63.2	100.7 ± 79.1	116 ± 66.4	0.53
Serum calcium (mg/dL)	8.99 ± 0.4	9.0 ± 1	9.1 ± 0.3	0.85
Serum 25OHD (ng/mL)	5.6 ± 8.1	8.4 ± 7.5	8.7 ± 6	0.49

One way analysis of variance was performed with post hoc analysis by Tukey's test.

*P* value < 0.05 was considered statistically significant.

**Table 3:** Frequency and distribution of plasma iPTH, serum calcium, and serum 25OHD levels in CaSR SNP, R990G genotypes.

Analytes	R990G polymorphism			*P* value
	AA (*n* = 112)	AG (*n* = 12)	GG (*n* = 16)	
Plasma iPTH (pg/mL)	90.4 ± 58.5	86.1 ± 58	148.7 ± 66.4	0.008
Serum calcium (mg/dL)	9.1 ± 0.3	9.1 ± 0.3	8.5 ± 0.4	0.002
Serum 25OHD (ng/mL)	8.3 ± 12.1	6.7 ± 7.1	11 ± 8.2	0.37

One way analysis of variance was performed with post hoc analysis by Tukey's test.

Tukey's test showed mean difference of iPTH and calcium levels were significantly different in GG genotype.

*P* value < 0.05 was considered statistically significant.

**Table 4:** Association of R990G:A986S haplotype with plasma iPTH, serum calcium, and serum 25OHD levels.

R990G:A986S	Frequency	Mean plasma iPTH difference pg/mL (95% CI)	*P* value	Mean serum calcium difference mg/dL (95% CI)	*P* value	Mean serum 25OHD difference (95% CI)	*P* value
A:G	0.68	0.00	—	0.00	—	0.00	—

A:T	0.17	15.3 (−7.06–37.64)	0.18	0.1 (−0.07–0.26)	0.25	−1.5 (−3.8–0.77)	0.2

G:G	0.12	49.4 (11.57–61.83)	0.0049	−0.11 (−0.29–0.08)	0.26	−0.9 (−3.63–1.69)	0.48

G:T	0.03	36.7 (0.48–98.4)	0.05	−0.51 (−0.88–−0.14)	0.0079	−1.6 (−7.24–3.96)	0.57

Haplotype association with quantitative variables was computed using online SNPstats software taking A:G haplotype as a reference.

*P* value < 0.05 was considered statistically significant.
